# Can MiR-503 be used as a marker in diabetic patients with ischemic stroke?

**DOI:** 10.1186/s12902-019-0371-6

**Published:** 2019-04-29

**Authors:** Saba Sheikhbahaei, Danesh Manizheh, Saadatnia Mohammad, Tajaddini Mohamad Hasan, Nazemian Saman, Rafiee Laleh, Motieian Mahsa, Amoushahi Khouzani Sanaz, Haghjooy Javanmard Shaghayegh

**Affiliations:** 1 Acquired Immunodeficiency Research Center, Isfahan University of Medical Sciences, Isfahan, Iran; 2 Student Research Committee, Isfahan University of Medical Sciences, Isfahan, Iran; 3 Isfahan Neurosciences Research Center, Isfahan University of Medical Sciences, Isfahan, Iran; 4 Applied Physiology Research Center, Isfahan University of Medical Sciences, Isfahan, Iran; 5 Mashhad University of Medical Sciences, Mashhad, Iran; 6 Isfahan University of Medical Sciences, Isfahan, Iran

**Keywords:** miR-503, MicroRNA, MiRNA, Ischemic stroke, Diabetes, Hyperglycemia, Ischemia

## Abstract

**Background:**

Some microRNAs are involved in diabetes pathology and some are known to have role in stroke. MiR-503 causes endothelial dysfunction in diabetic patients, predisposing to ischemia. There has been no study evaluating Mir-503 level in diabetic patients with or without ischemic stroke.

**Methods:**

We designed a cross-sectional study to assess and compare serum level of MiR-503 in 4 groups of diabetic patients with ischemic stroke (I), non-diabetic patients with stroke (II), diabetic patients (III), and healthy controls (IV) in acute phase and 3 months later.

**Results:**

Our data analysis showed that mean relative expression of MiR-503 in group (I) was significantly higher than 3 other groups (*p* < 0.05). The level of miR-503 was related to the patients’ fasting blood glucose, Cholesterol level, NIHSS score and acute–phase modified Rankin Scale (mRS) (r = 0.49, *p* = 0.001, r = 0.5, *p* = 0.009, r = 0.45, *p* = 0.009, r = 0.48, *p* = 0.003, CI = 95%). Relative expression of miR in patients with mRS ≤ 2 (good outcome) was lower than in patients with mRS > 2 (poor outcome) (*p* = 0.008). After 3 months, level of miR decreased significantly only in group (I) (*p* = 0.002). Mean relative expression of miR-503 in chronic phase was not significantly different among groups (*p*-value> 0.05). There was no relation between miRNA level and mRS in chronic phase.

**Conclusion:**

Hyperglycemia and ischemia together raise the level of MiR-503 acutely but it does not remain at high level after 3 months. Although higher miR was related to more disability in acute phase, it does not affect long-term outcome in ischemic patients. As MiR-503 is stable enough in blood it can be used as a potential diagnostic marker of an ischemic stroke in diabetic patient. Its level also is an indicator of stroke severity and patients’ short-term outcome. It is recommended to study whether antagomiR-503 is a new therapeutic agent reducing the severity of and disability due to stroke.

## Background

Ischemic stroke accounts for 85% of strokes [1]. Its incidence rate and the complications have increased in previous decades [2]. Diabetes is a recognized independent risk factor of stroke. Hyperglycemia causes oxidative stress, leading to endothelial dysfunction and finally microvascular complications. The latter is the main cause of ischemic stroke [3]. Hyperglycemia confers greater recurrence rate and affects patients’ outcome as well [4, 5]. Diabetic patients with ischemic stroke are associated with poorer prognosis and higher mortality [5]. Stress hyperglycemia in non-diabetic patients after an ischemic stroke increases in-hospital mortality rate and diminishes functional recovery [6].

MicroRNAs are non-coding, tiny, 20–25 nucleotides-long RNAs; which bind to 3’UTR of the target mRNA leading to mRNA degradation or translation inhibition [7, 8]. The role of microRNAs in progression of diabetes and its complications including micro- and macrovascular dysfunction has been highlighted [9]. Downregulation of MiR-126 inhibits proliferation and migration of endothelial progenitor cells [10] while expression of miR-375 plays a regulatory role in pancreatic cell proliferation and insulin secretion [11]. Studies also revealed bidirectional role of miRNAs in acute ischemic stroke pathology; neuroprotective and neuro-damaging. Therefore, two therapeutic strategies are suggested for patients with acute ischemic stroke to decrease the burden of disease; knocking down neuro-damaging miRNAs by antagomirs which neutralize the function of miRNA [9, 12–14] and discovery of neuroprotective miRNA mimics [15].

A study revealed that expression of miR-503 was significantly increased in ischemic muscles of diabetic mice and also diabetic patients undergoing foot amputation for critical ischemia. It has shown that miR-503 impairs reparative angiogenesis after limb ischemia in diabetes mellitus (DM). As evidence, inhibition of miR-503 normalizes post-ischemic blood flow and improves recovery by neovascularization in diabetic mice [16]. There is no study evaluating miR-503 expression in diabetic patients with ischemic stroke. Therefore we designed this study to see how does miR-503 level change in a diabetic patient when an ischemic stroke occurs and 3 months later. We aimed to find whether it can be used as a diagnostic marker.

## Methods

This cross-sectional study was conducted in Alzahra hospital, Isfahan, Iran, 2015–2017. The medical ethics committee of Isfahan University of Medical Sciences approved the study. Study was designed to compare miR-503 level in 4 groups of diabetic patients with stroke (I), non-diabetic patients with stroke (II), diabetic patients without stroke (III) and healthy controls. Sample size for case groups was calculated to be 15 patients in each group based on Caporali et al. study [16]. Cases of groups (I) and (II) were selected consecutively from patients with ischemic stroke who were admitted to the department of neurology of Alzahra hospital within 72 h after symptom onset. Diabetic patients who were diagnosed according to the WHO diabetes mellitus diagnostic criteria at the health clinic of Alzahra were randomly chosen for group (III). Informed consent was obtained from participants or their next of kin if they were not conscious. Patients with any of the disease which may affect the plasma level of miR-503 including history of GI and CNS tumors, pulmonary, neurodegenerative, cardiovascular, autoimmune diseases and previous stroke within prior year did not meet inclusion criteria. Patients with ischemic stroke who were affected by post-ischemic cerebral hemorrhage or cardiac attack during their hospital stay were excluded from further analysis. Diagnosis of acute ischemic stroke was based on clinical findings and confirmed with CT/MRI scans. The extent of impairment due to stroke is measured objectively by National Institutes of Health Stroke Scale, (NIHSS). NIHSS Scoring is classified as: (0) no stroke symptom, [1–4] mild, [5–15] moderate, [16–20] moderate to severe and [21–42] severe stroke [17]. Degree of disability in daily activities is measured by modified Rankin Scale (mRS) which runs from 0 to 6, perfect health without symptom to death. Patients with mRS ≤2 in acute phase were considered to have good outcome [16]. Patients were followed for a period of 3 months and long-term outcome was also evaluated by mRS again. Stroke subtypes were classified according to TOAST classification into large-artery atherosclerosis, small vessel occlusion, cardioembolism and stroke of undetermined etiology [18].

From each participant, 5 ml of whole blood was collected into an EDTA-containing tube (BD Vacutainer, Plymouth, UK) by venipuncture. Blood samples of group (I) and (II) were obtained in the first 3 days from the onset of symptoms. Blood samples were fractionated by a centrifuge at 3000 g for 15 min at 4 °C. The plasma layer was then aliquoted and stored at − 70 °C. Total RNA containing miRNAs was extracted from samples using the Qiazol reagent followed by miRNeasy mini kit according to the manufacturer protocol. Reverse transcription reactions were performed with 1 μg total RNA using miScript II RT kit (Qiagen, Germany) after DNase I treatment (Ambion, USA) according to manufacturer protocol.

Real time quantitative RT-PCR was performed using ABM EvaGreen miRNA qPCR Mastermix according to the manufacturer instructions. Relative quantification was achieved by normalization to the amount of U6. All reactions were performed in triplicate. All predesigned primers for miR-503 and U6 were purchased from ABM (Applied Biological Materials, Canada). The relative gene expression levels were calculated using Relative Expression Software Tool (REST) software version 2009 (QIAGEN, Germany). Fasting blood sugar (FBS), HbA1C, cholesterol and triglyceride were recorded from all individuals. MiR level was compared in acute and chronic phase among patients by paired t-test. Independent T test and ANOVA were used for quantitative variables and chi-square was applied in qualitative variables after using Kolmogorov-Smirnov in SPSS version 22. For variables, which were not normally distributed, nonparametric tests were used. All the analyses were conducted in a blind mode.

## Results

**Fig. 1:**
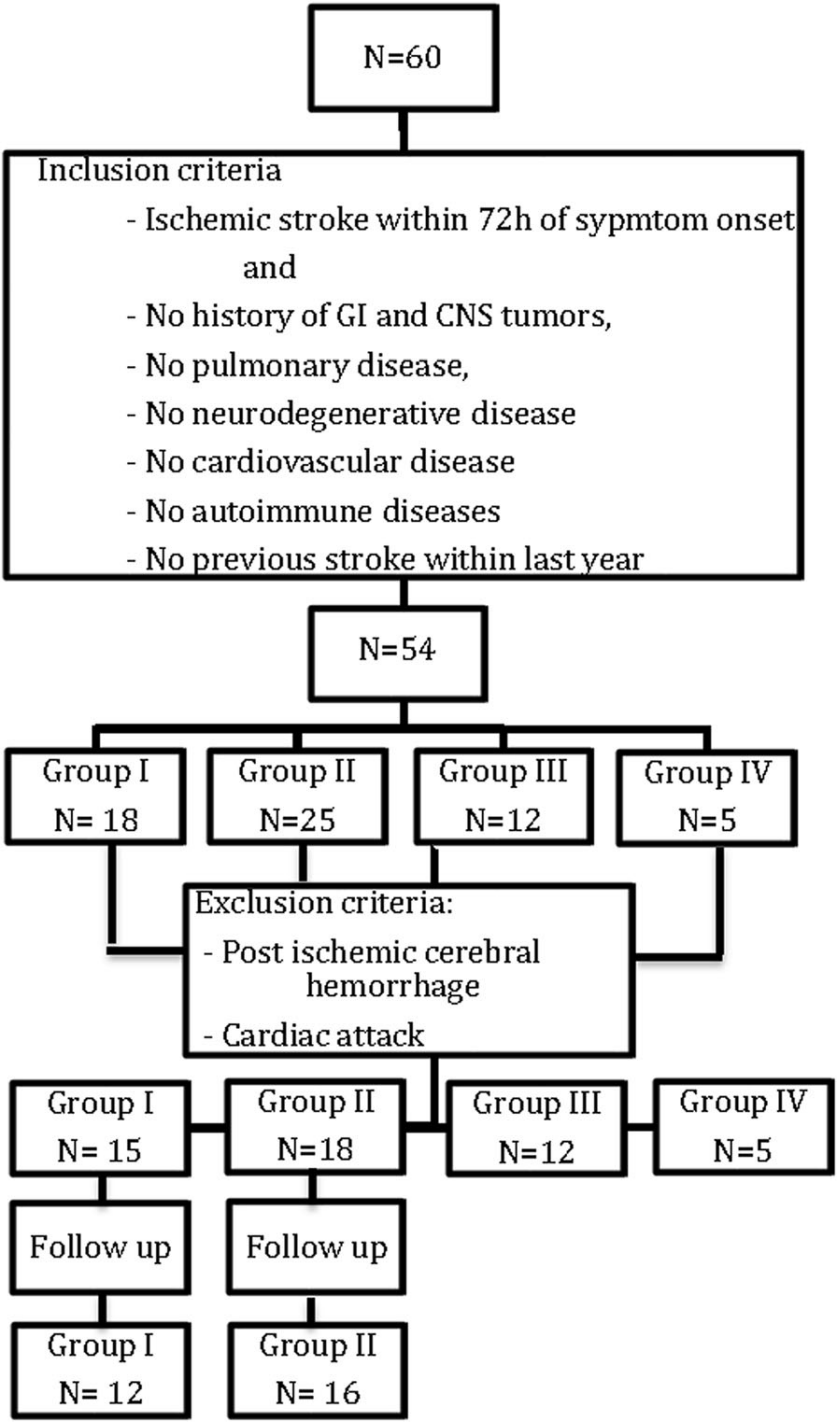
The diagram shows the number of patients included in the study based on the inclusion and exclusion criteria

Sixty patients entered the study and allocated in 4 groups: 18 diabetic patients with stroke (I), 25 non-diabetic patients with stroke (II), 12 diabetic patients without stroke (III) and 5 healthy controls. Six patients did not fulfill the inclusion criteria (have a simultaneous GI cancer, Crohn’s disease, Scleroderma, heart attack and previous stroke in prior year), 4 were excluded due to post stroke hemorrhage, cardiac arrest and a newly diagnosed colorectal cancer. Finally, 50 patients participated in our investigation, 15 patients group (I), 18 patients group (II), 12 patients group (III) and 5 healthy individuals. Three patients died during the period of follow up and 2 other patients did not come back to visit for unknown reason. Therefore, we could collect samples of 12 patients from group (I) and 16 patients from group (II) after 3 months (Fig. 1).

Mean age and sex distribution were not different among patients in 4 groups (*p* > 0.05). HbA1C was not significantly different between group (I) and (III). FBS level was greater in group (I) than group (II) and (IV). Serum cholesterol (Chol) and triglyceride (TG) level did not statistically differ among groups (Table 1).

**Table 1:** Distribution of sex, mean age, fasting blood glucose (FBS), glycemic control (HbA1C), triglyceride (TG) and Cholesterol in all groups

	N	Age (mean ± SD)	Sex (F/M)	FBS	HbA1C	TG	Chol
Group (I)	15	64.4 ± 12.6	10/5	194 ± 54	7.43 ± 0.9	186 ± 108	196 ± 51
Group (II)	18	71.4 ± 10	10/8	97 ± 16	–	126 ± 58	144 ± 25
Group (III)	12	65.5 ± 16	7/5	162 ± 11	7.2 ± 0.8	157 ± 29	184 ± 45
Control (IV)	5	68.2 ± 6	3/2	90 ± 9	–	118 ± 25	154 ± 20

Mean relative expression of miR-503 was 3.29 ± 2 in group (I), 1.88 ± 1.4 in group (II), 2.49 ± 1.8 in group (III) and 1.1 ± 0.4 in control group. Relative expression of miR-503 was significantly different among groups (*p*-value = 0.04). MiR-503 level was higher in diabetic- stroke patients in comparison with stroke only patients and control individuals (*p*-value = 0.02, p-value = 0.01). The study was conducted in 29 females and 21 males. Mean relative expression of miR503 in males was significantly higher than females (3.3 ± 2 vs. 1.9 ± 1.3, *p*-value = 0.01).

Our results showed association between the level of miR-503 and FBS, Chol, NIHSS score and acute–phase mRS (r = 0.49 p-value = 0.001; r = 0.5 *p* = 0.009; r = 0.45 *p* = 0.009; r = 0.48 *p* = 0.003 CI = 95%) but no correlation was seen between the level of miR-503 and FBS, Chol, mRS in acute–phase and NIHSS score of patients in groups (I), (II) and (III) separately.

Relative expression of miR-503 differs significantly in patients with different stroke severity according to NIHSS scoring (*p* = 0.02). The expression levels were 1.9 ± 1.2 and 3.9 ± 2.7 in patients with moderate stroke and moderate to severe stroke respectively (*p* = 0.01). There was no significant difference in FBS, TG and Chol level among patients with different stroke severity. Relative expression of miR in patients with mRS ≤ 2 (good outcome) was 1.3 ± 0.5 and in patients with mRS > 2 (poor outcome) was 2.9 ± 1.5 in acute phase (*p* = 0.008).

In the 2nd sampling, 3 months later, mean relative expression of miR-503 in diabetic patients with stroke and non-diabetic patients with stroke was 1.23 ± 0.6 and 1.4 ± 0.4 respectively. Relative expression of miR-503 in chronic phase was not significantly different among groups (*p*-value> 0.05). It has been decreased significantly in group (I) (*p* = 0.002) and no statistical change in stroke only patients. Patients of group (I) had mRS score of 3.8 ± 1.2 in acute phase and 2 ± 1.5 in chronic phase (*p* = 0.01). Relative expression of miR in patients with mRS ≤ 2 was not different from patients with mRS > 2 in chronic phase. Mean FBS level did not differ in diabetic patients after 3 months.

Patients in all groups were not different regarding history of hypertension, hyperlipidemia, antihypertensive or antiplatelet drug history and clinical manifestation. Mean relative expression of miR-503 did not differ in patients with different clinical manifestations and various stroke subtypes. Expression of miR-503 was not different in patients with different ejection fractions. It was also equal among patients without carotid stenosis and patients with partial or total occlusion (Table 2).

**Table 2:** miR-503 expression and mRS in acute and chronic phase, NIHSS score, history of hypertension and hyperlipidemia, using antihypertensive and antiplatelet drugs, different clinical manifestations and stroke subtypes in all groups

	Group (I)	Group (II)	Group (III)	Control	
miR-503 expression (acute)	3.3 ± 2.1	1.9 ± 1.5	2.5 ± 1.8	1.1 ± 0.4	*P* = 0.045
					(I), (II): p = 0.02, (II), (III): *p* = 0.3
					(I), (III): *p* = 0.2 (II), (IV): *p* = 0.4
					(I). (IV): *p* = 0.016 (III), (IV): *p* = 0.1
miR-503 expression (chronic)	1.2 ± 0.6	1.3 ± 0.4	2.5 ± 1.8	1.1 ± 0.4	*P* = 0.009
					(I), (II): *p* = 0.8, (II), (III): *p* = 0.005
					(I), (III): *p* = 0.004 (II), (IV): *p* = 0.7
					(I), (IV): *p* = 0.8
NIHSS score	12.2 ± 5.2	11.8 ± 5.3	–	–	*p* > 0.05
mRS acute	3.4 ± 1.2	3.3 ± 1.3	–	–	*p* > 0.05
mRS chronic	2 ± 1.5	3.2 ± 1.9	–	–	*p* > 0.05
Hypertension (Y)	11 (84.6%)	13 (72.2%)	3 (25%)	2 (40%)	*p* > 0.05
Hyperlipidemia (Y)	5 (38.5%)	9 (50%)	4 (33.3%)	2 (40%)	*p* > 0.05
Antihypertensive drug	10 (66.6%)	13 (72.2%)	3 (25%)	2 (40%)	*p* > 0.05
Antiplatelet drug	6 (46.2%)	11 (47.8%)	–	–	*p* > 0.05
Clinical manifestation			–	–	*p* > 0.05
Altered consciousness	2 (13.3%)	7 (38.8%)			
Diplopia/dysarthria/facial paralysis	10 (76.9%)	8 (44.4%)			
Hemiparesis/hemisensory deficit	10 (76.9%)	13 (72.2.%)			
Stroke subtype					*p* > 0.05
LVA	6 (40%)	10 (52.6%)			
SVA	8 (53.3%)	6 (31.6%)			
Cardioembolic	1 (6.7%)	3 (15.8%)			

## Discussion

Many miRNAs have identified role in stroke emerging. A mice model trial showed post-ischemic increase in miR-200c level resulted in brain cells death [19]. Another study demonstrated that antimiRNA reduces stroke-induced brain damage and lower expression of the correspondent miRNA is related with less neurological deficit [20]. Expression of pro-inflammatory cytokines and chemokines and consequently brain damage is reduced by inhibition of miR-210 in acute phase of ischemic stroke. Conversely, an in vitro study elucidated high level of miR-210 under hypoxemic situation induces angiogenesis and neurogenesis [21]. Altered expression of miR-124 in cerebral ischemia also suggests promotion of neuronal survival in ischemic condition [22].

Peripheral blood samples of patients with acute ischemic stroke showed elevated miR-223 and miR-145 level compared to controls [23, 24]. However some studies reflected that miR is a repair biomarker and higher levels are accompanied with better outcome [25, 26]. It has been suggested that miRNA has a diagnostic and prognostic value for ischemic stroke [27, 28]. A therapeutic potential seems to be present by neutralizing miRNAs that cause neuronal death and prevent regeneration [29, 30].

One of the major risk factors for ischemic stroke is diabetes. MiRNA alterations in vascular endothelial cells (EC) increase stroke incidence in diabetic patients. There is only one recent animal study assessing miR-503 changes in ischemic stroke [31]. Our study is the first human investigation on miR-503 expression in cerebral ischemia plus high glucose state. We observed higher level of miR-503 in diabetic patients with stroke (group (I)) compared to stroke only patients (group II) suggesting that hyperglycemia and ischemic situation cause overexpression of miR-503. On the other hand there was no significant difference in miR expression level among non-diabetic patients with stroke (group (II)) and diabetic only patients (group (III)) compared to control subjects. Therefor ischemia or hyperglycemia do not induce adequate change in miR-503 expression separately. The study was designed in 2 steps with the interval of 3 months, in order to compare the expression of miR-503 in acute and chronic phase. Chronic miR level in group (I) decreased to the miR level in group (II). This shows miR-503 is a biomarker of acute phase and falls to normal range by 3 months.

Caporali et al. has studied the regulation of miR-503 expression in hyperglycemic condition and observed that miR-503 is upregulated in plasma of diabetic rats with critical limb ischemia. They enhanced the expression of miR-503 using lentiviruses and found inhibited EC proliferation, migration, and network formation [32]. It has been studied that miR-503 affect cell cycle arrest through degradation of CDC25A in response to TGF-β [33]. In ECs, MiR-503 directly downregulates CCNE1 and cdc25A in a situation mimicking ischemia and diabetes (high glucose/low growth factors). It has been demonstrated that miR-503 is involved in diabetic endothelial dysfunction [16].

We explored possible association between miRNA levels and mRS, and NIHSS too. Higher level of miR is detected in patients with more disability on daily activity. Also relative expression of miR-503 was lower in patients with good outcome (mRS ≤2) rather than poor outcome (mRS > 2). A microRNA profiling in ischemic stroke patients showed many microRNAs are downregulated in good outcome (mRS < 2) stroke patients compared to normal controls, irrespective of stroke subtype [28]. We can hypothesize that miR-503 increases inflammation and oxidative stress, which is associated with increased risk of stroke and disability in diabetic patients. We observed patients with moderate to severe stroke showed greater level of miR as well. Since miR-503 level correlates with NIHSS score, it is suggested as a neurodamaging factor. MiR-223 was also found to have positive correlation with the severity of stroke [34].

We also aimed to know how miR level alterations affect patients’ recovery few months after stroke. As mentioned previously, no correlation was found between initial miR level of the patients and their mRS in chronic phase. However, It has been shown that miR-503 might suppress post-ischemic neovascularization in diabetes mellitus. Also neutralizing miR-503 activity improved vascular healing and blood perfusion to ischemic limb [16, 32]. So we expected to see more disability remaining after 3 months in patients who had higher initial expression of miR but we did not find relation between miR level and patients’ long-term outcome.

While searching about the mechanism supporting our data, we found that in ECs exposed to high glucose, transcription of miR-503 increased and negatively affected pericytes’ function [35]. In addition, miR-503 targeting E2F3, inhibits proliferation and induces apoptosis by cell cycle arrest in G0/G1 [36]. It has been recently discovered that losartan improves diabetic nephropathy by inhibiting miR-503 and there is a hypothesis that overexpression of miR-503 is one of the causes for diabetic nephropathy [37]. This finding is evidence for the role of miR-503 in diabetes related microvascular dysfunction. Another study revealed that miR-503-3p induces apoptosis in vascular smooth muscle cells [38, 39]. As our investigation is a cross-sectional study no causality relationship could be interpreted. We only observed that miR-503 is remarkably increased in diabetic patients for whom ischemic stroke has occurred but evidences brings up the hypothesis that miR-503 may have a role in pathogenesis of ischemia in hyperglycemic state. It has been shown that fenofibrate and Brassica Oleracea,which remarkably lower the level of miR-503 in brain circulation, reduce stroke occurrence in mice., In-vitro overexpression of microRNA-503 in endothelial cells decreases cellular viability [31].

A similar study researched about miR role in acute ischemic stroke in diabetic patients showed that miR-223 and miR-246a are downregulated in hyperglycemia. This precipitates platelet activation and increases the risk of stroke. It has also suggested that platelet miRNA-223 and miR-146a are specific markers for diabetes mellitus but not for ischemic stroke [40]. Downregulation of miR-223 in plasma and platelet plus overexpression of miR-144 increased susceptibility to ischemic stroke in diabetic patients [41]. A study has found that miR29a is downregulated in simulated ischemia plus hyperglycemia and leads to angiogenesis [42].

Tan et al found miRNAs expression profile varies among different subtypes of ischemic stroke [28, 43]. We didn’t discover any difference in miR-503 expression among patients with large artery atherosclerosis, small vessel occlusion, cardioembolism and undetermined but as our sample size was not large enough to do analysis in subgroups we could not rely on this finding. One of the most important limitations of our study was the small sample size. As this was the first study on this topic in human beings and only few supportive studies were conducted, It was not eligible for greater amount of funding. For this it is highly suggested to repeat this study in a larger number of patients to see whether the results are confirming. Another drawback in our study was including all diabetic patients, better to restrict only newly diagnosed diabetic patients.

## Conclusion

miR-503 is significantly overexpressed in diabetic patients with acute ischemic stroke and is stable enough in the blood to develop as potential diagnostic marker. We could not conclude that MiR-503 is involved in pathogenesis of stroke but further studies should be done to evaluate the hypothesis that miR-503 induces apoptosis and renders inflammatory response leading to ischemia. MiR level is also related to stroke severity and patients’ short-term outcome. So antagomiR-503 may be a new therapeutic agent reducing severity and disability of stroke.

## Abbreviations

CCNE1: cyclin E1
CDC25A: cytokine-derived cell cycle
CDK: cyclin-dependent kinase
EC: endothelial cell
miR-503: microRNA-503
mRS: modified Rankin Scale
NIHSS: National Institutes of Health Stroke Scale
TOAST: Trial of Org 10,172 in Acute Stroke Treatment
